# Psychometric Assessment of a New Pain-Specific Patient-Reported Outcome Measure for Pelvic Floor Surgery Using Exploratory Factor Analysis

**DOI:** 10.1007/s00192-026-06620-9

**Published:** 2026-04-16

**Authors:** Sheymonti S. Hoque, Susannah Ahern, Helen E. O’Connell, Rasa Ruseckaite

**Affiliations:** 1 School of Public Health and Preventive Medicine, Monash University, 553 St Kilda Rd, Melbourne, VIC 3004 Australia; 2 Department of Surgery, University of Melbourne, Melbourne, VIC Australia

**Keywords:** Pain, Pelvic floor disorder, Pelvic floor procedure, Patient-reported outcome measure, Psychometric assessment, Exploratory factor analysis

## Abstract

**Introduction and Hypothesis:**

Pelvic floor procedures are associated with pain in some women, adversely affecting health-related quality of life (HRQoL). Current patient-reported outcome measures (PROMs) are inadequate for assessing pain following pelvic floor procedures. A new 16-item pain-specific PROM, the Pelvic Floor Procedure Pain Questionnaire (PPPQ), was developed to address this gap. This study aimed to undertake a psychometric assessment of the PPPQ through field testing and to understand its latent structure.

**Methods:**

The PPPQ was administered online to 103 adult women from pelvic mesh support groups who underwent pelvic floor surgery. Exploratory factor analysis (EFA) using principal axis factoring and Promax rotation was conducted to assess the PROM’s psychometric properties (i.e. structural validity, internal consistency), refine items, and explore the relationship between items.

**Results:**

Most commonly, participants were aged 60–69 years (*n* = 46, 44.7%) and from the United Kingdom (*n* = 48, 46.6%). EFA identified a 3-factor model comprising: (1) HRQoL disruptions from post-pelvic floor procedure pain, (2) characteristics and management of post-pelvic floor procedure pain, and (3) pain avoidance behaviours, resulting in a refined 11-item PPPQ. The PPPQ and its items demonstrated acceptable internal consistency (Cronbach’s *α* > 0.70), with items clustering into expected pain-related themes confirming structural validity.

**Conclusions:**

Study findings suggest that the revised, 11-item PPPQ has good reliability (internal consistency) and structural validity. While the psychometric evaluation demonstrates its suitability in assessing pain post-pelvic floor surgery, additional psychometric testing is needed. Full development of the PROM could offer deeper insights into pain and help monitor health outcomes in this population.

**Supplementary Information:**

The online version contains supplementary material available at 10.1007/s00192-026-06620-9.

## Introduction

Pelvic floor procedures are a common treatment for pelvic floor disorders (PFDs), including stress urinary incontinence (SUI) and pelvic organ prolapse (POP) in women, particularly when conservative approaches such as lifestyle modifications and pelvic floor physiotherapy fail to provide adequate symptom relief or functional improvement [1]. Surgical interventions include transvaginal native tissue repairs, transvaginal mesh repairs, and abdominal approaches (open or laparoscopic) using mesh or native tissue [2]. These procedures aim to provide anatomical correction, symptom relief, and improvements in bladder and bowel function, contributing to improved quality of life [2]. However, like all surgeries, pelvic floor procedures carry risks and complications, including recurrence of incontinence or prolapse, infection, and haemorrhage [3]. Pain is a particularly significant complication following pelvic floor surgery, mainly associated with mesh complications [3]. Pain is a key health-related quality of life (HRQoL) concern, as it can severely affect daily functioning, mobility, and psychosocial well-being in women after pelvic floor surgery [4]. Data captured between 2021 and 2024 by the Australasian Pelvic Floor Procedure Registry reveal that among 91 Australian women who presented with symptoms before mesh excision or revision surgery, pain was the most reported symptom (67.0%) [5].

Despite its impact, pain and the patient experience of pain are often under-recognised and poorly assessed by existing patient-reported outcomes measures (PROMs), which are designed to capture patients’ perspectives without clinical input [6, 7]. Several studies have identified the need for a pain-specific PROM for women following pelvic floor procedures [4, 6, 8–10]. In response, we developed the Post-Pelvic Floor Procedure Pain Questionnaire (PPPQ), a new pain-specific PROM for women with PFDs postsurgery [11, 12]. The development of the instrument was guided by the 2022 US Food and Drug Administration ‘Principles for selecting, developing, modifying, and adapting patient-reported outcome instruments for use in medical device evaluation’, which outlines current best practices for patient-reported outcome (PRO) instrument design and validation [7]. In this study, we aimed to undertake a psychometric evaluation of the PPPQ through field-testing and uncover the underlying structure of the instrument using an exploratory factor analysis (EFA). A formal hypothesis for the study was not adopted owing to the nature of the EFA.

## Materials and Methods

### PROM Development

**Fig. 1:**
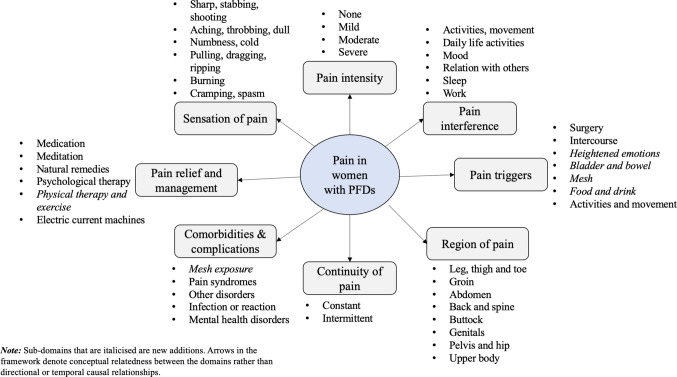
Conceptual framework highlighting important attributes of pain associated with pelvic floor surgery in women [11]

After establishing the need for a new instrument, a pool of items (*n* = 114) was drafted under eight pain-related domains of a conceptual framework that was developed on the basis of the findings from qualitative interviews [4, 11] and a previously conducted scoping review of the literature [6]. This framework (Fig. 1) conceptualised postoperative pain from pelvic floor surgery as a multidimensional experience, covering aspects including sensations, location of pain, triggers, impact, continuity, and intensity. The framework was developed to support item generation and content validity, rather than to represent a causal or disease progression model. Further information on the framework is detailed in our mixed-methods paper [11].

Of the 114 draft items, 35 were generated through a Delphi process with clinicians and patients [11]. Focus group discussions/interviews involving women with lived experience of pain following pelvic floor surgery were conducted to pre-test the 35 candidate items for clarity and relevance, leading to the removal of 19 items. Following the pre-testing, a preliminary version (first draft) of the PROM (the PPPQ) was produced, covering 16 items under seven domains: (1) region of pain, (2) pain triggers, (3) sensation of pain, (4) intensity and continuity of pain, (5) pain interference, (6) comorbidities and complications, and (7) pain relief and management [12]. The PPPQ is specifically designed to capture pain across the postoperative continuum following pelvic floor surgery, including acute postsurgical pain and persistent or chronic pain. Given the multifactorial nature of post-procedural pelvic pain, the PROM captures pain experiences that may reflect nociceptive and/or neuropathic features, without aiming to diagnostically differentiate pain mechanisms. The instrument is intended for use in clinical care settings, including specialist clinics and hospitals.

### Description of Items in the PPPQ

As described earlier, the PPPQ comprised 16 items under seven pain-related domains. A combination of response formats, including ‘tick all that apply’ and Likert-type scales. Items which have the ‘tick all that apply’ format consist of Q1 (domain 1), Q2 (domain 2), Q4 (domain 3), Q8 (domain 5), Q13 (domain 6), and Q15 (domain 7). These items were converted into binary variables (yes = 1, no = 0) for each listed option, and treated as count variables representing the total number of options ticked. For these domains, summated scores were intended to reflect the breadth of pain experiences endorsed within a domain rather than pain severity. Likert-type items (e.g. frequency, intensity, impact) were Q3 (domain 2), Q5–7 (domain 4), Q9–12 (domain 5), Q14 (domain 6), and Q16 (domain 7). These items were coded numerically 0 to 4 or 1 to 5, with higher values indicating greater symptom severity or impact.

### Study Design

This was a prospective field-testing study to test the psychometric properties and understand the latent structure of the PPPQ. Field-testing involved adult women (≥ 18 years) with a PFD who had undergone surgery for stress urinary incontinence (SUI) and/or pelvic organ prolapse (POP). Eligible participants were required to complete a brief set of screening questions before starting the PROM (see Supplementary Material 1).

### Sample Size

The sample size for the study was calculated using the sample-to-item ratio of 5:1, recommended by Gorsuch [13] and Hatcher [14]. The 5:1 ratio is considered acceptable in practice. Our PROM comprised 16 items, and based on the ratio, the estimated target sample size was 80. While Gorsuch [13] is sometimes cited as recommending a 10:1 ratio, the original text specifies a minimum of five participants per item. Hatcher [14] also noted that a sample of at least 100 is desirable when feasible, and Kline [15] recommended an absolute minimum of 100 participants. Our PROM comprised 16 items, and the estimated target sample size was 80 based on the 5:1 ratio. The final sample size, however, exceeded this target and met Kline’s recommended minimum [15].

### Participant Recruitment

Recruitment was conducted via international online pelvic mesh support groups, identified from Google and Facebook searches. These included Sling The Mesh (United Kingdom and Northern Ireland), Rectopexy Support Group (United Kingdom), Mesh Down Under Support Group and Surgical Mesh Support Network from New Zealand, and the Australian Pelvic Mesh Support Group. A study invitation letter and advertisement were posted within these groups. The study was also advertised on other social media platforms (i.e. Instagram, LinkedIn, X.) by mesh support group founders/administrators. The study’s explanatory statement was embedded within the PROM itself. Interested participants completed the PROM anonymously via Qualtrics, accessed through a link or by scanning a QR code. Before commencing the PROM collection, participants were required to complete the screening questions, which appeared in a section in Qualtrics before the PROM. Women were considered eligible for the study given their participation in the mesh supports and their ability to complete the screening questions. Participants were not reimbursed after completing the PROM. Recruitment occurred between October 2024 and February 2025. A flow diagram outlining the PROM administration process is provided in Supplementary Material 2. All PROM responses were exported from Qualtrics into Microsoft Excel for analysis.

### Statistical Analysis

Descriptive statistics were calculated to summarise participant characteristics. The psychometric properties of the instrument were assessed through an EFA [15], which was used to identify ‘factors’ (underlying dimensions) among the items [16, 17]. Guided by the conceptual framework (Fig. 1), we hypothesised that the PROM is a multidimensional measure. This informed our EFA, including the selection of the rotation method. Although the Consensus-based Standards for the Selection of Health Status Measurement Instruments (COSMIN) recommends a confirmatory factor analysis (CFA) for testing pre-defined factor structures, EFA was deemed appropriate at this early stage to explore how pain-related items clustered empirically, rather than to test a pre-defined model separating sensory and affective pain domains [16].

Before conducting the EFA, we checked the suitability of the data to ensure factorability and the reliability of the analysis. This assessment included consideration of the sample size, continuous variables, the Kaplan–Meyer–Olkin (KMO) measure for sampling adequacy test and Bartlett’s test of sphericity, and factorability of the correlation matrix [17]. Following conventional criteria, a KMO value of ≥ 0.60 was considered acceptable for sampling adequacy, and a significant Bartlett’s test of sphericity (*p* < 0.05) indicated that the correlation among items was sufficient to proceed with the EFA [17].

Factors for the EFA were extracted using principal axis factoring, as our data was not normally distributed [17, 18]. The number of factors to retain in the final model was determined using four investigative approaches [17, 19, 20]. These included:

- Method 1—Parallel analysis: This statistical method compares the real data to a generated random set of data. In this analysis, we retained only factors that were stronger than those from the random data.
- Method 2—Kaiser criterion/eigenvalue cutoff rule: The most common method used to determine the number of factors to retain. As suggested by the Kaiser criteria, factors with an eigenvalue > 1 were retained [20].
- Method 3—Visual scree test: Graphical method which involves plotting eigenvalues against factor number (x-axis), and finding where the line starts to level off (‘elbow’) on a plot.
- Method 4—Running EFA iteratively: running the factor analysis iteratively with rotation and removing items that demonstrated poor factor loadings and were problematic, then repeating the process until a simple structure was obtained. This is a common practice in item development and refinement as it is exploratory and the decisions made are driven by data but guided by theory [21].

To ensure factors were easy to interpret, the factors were rotated using the Promax ‘oblique’ rotation method, which allowed factors to correlate with each other [16]. During the analysis, any missing PROM response data were replaced using mean imputation, where the mean of each item was substituted for missing responses. Mean imputation was applied to retain the full sample for factor analysis as there was a small percentage of missing values per item, which appeared random.

Before finalising the factor solution, items were retained if they loaded ≥ 0.30 on at least one factor and if the difference between their primary and secondary loadings was ≥ 0.20. As suggested by Tabachnick and Fidell [22], items that loaded ≥ 0.32 on two or more factors were considered cross-loading and were reviewed for potential removal. Communalities (h^2^) below 0.30 were also examined as indicators of weak contribution to the factor solution, with decisions informed by the conceptual relevance of the item.

All analyses were conducted in SPSS version 29.

#### Psychometric Evaluation

The COSMIN checklist was used to guide the evaluation of the new PROM’s psychometric properties [16]. Of the nine properties included in the checklist, two were assessed in this study—structural validity and internal consistency—based on the type of factor analysis performed [16]. In line with COSMIN guidelines, EFA was conducted as the initial step in the psychometric evaluation [16].

Structural validity was assessed by examining the underlying structure of the items and identifying latent factors through EFA [16]. Structural validity was further assessed using factor correlation analysis and communality values (i.e. the proportion of variance in an item explained by the extracted factors) [16]. Internal consistency was assessed using Cronbach’s alpha (*α*), with values ≥ 0.70 considered acceptable for research purposes [23].

## Results

### Participant Characteristics

One hundred forty-nine women participated in the study, of which 103 completed the PROM. Most of the women were aged between 60 and 69 years (*n* = 46, 44.7%) and were from the United Kingdom (*n* = 48, 46.6%) (Table 1). Forty (38.8%) women were from Australia and New Zealand. Ninety-eight (95%) women experienced pain post-pelvic floor procedure, and 55 (53.4%) reported developing pain within one week postsurgery, which is most likely to be temporary postoperative pain (Table 1).

**Table 1:** Characteristics of participants included in the EFA (*n* = 103)

Variable	Category	*n* (%)
Age (years)	30–39 40–49 50–59 60–69 70+ Not stated	3 (2.9) 9 (8.7) 31 (30.1) 46 (44.7) 13 (12.6) 1 (1.0)
Pain postsurgery	Yes No	98 (95.1) 5 (4.9)
Onset of pain postsurgery	Within 1 week Within 1 month Within 6–12 months After > 1 year Not applicable*	55 (53.4) 14 (13.6) 13 (12.6) 16 (15.5) 5 (5.8)
Country	United Kingdom Oceania^ Europe North America Africa Not known	48 (46.6) 40 (38.8) 5 (4.9) 2 (1.9) 1 (1.0) 7 (6.8)

^*^Reflects participants who responded ‘no’ to postsurgical pain

^Oceania: Australia and New Zealand

### Exploratory Factor Analysis

The sample size for the study was adequate for EFA, exceeding the calculated target sample size and the minimum recommended size for the analysis [15]. The dataset contained both continuous and ordinal variables. The KMO statistic of 0.796 exceeded the minimum adequacy threshold (> 0.60), indicating factorability. The Bartlett’s test of sphericity indicated that the correlation matrix was not random (*p* < 0.001). The correlation matrix showed that most item correlations were ≥ 0.30, indicating sufficient relatedness for EFA. The determinant (0.014) exceeded the 0.00001 threshold, confirming matrix suitability for factor analysis [17]. The SPSS outputs for KMO, Bartlett’s test, and the correlation matrix are shown in Supplementary Material 3.

**Fig. 2:**
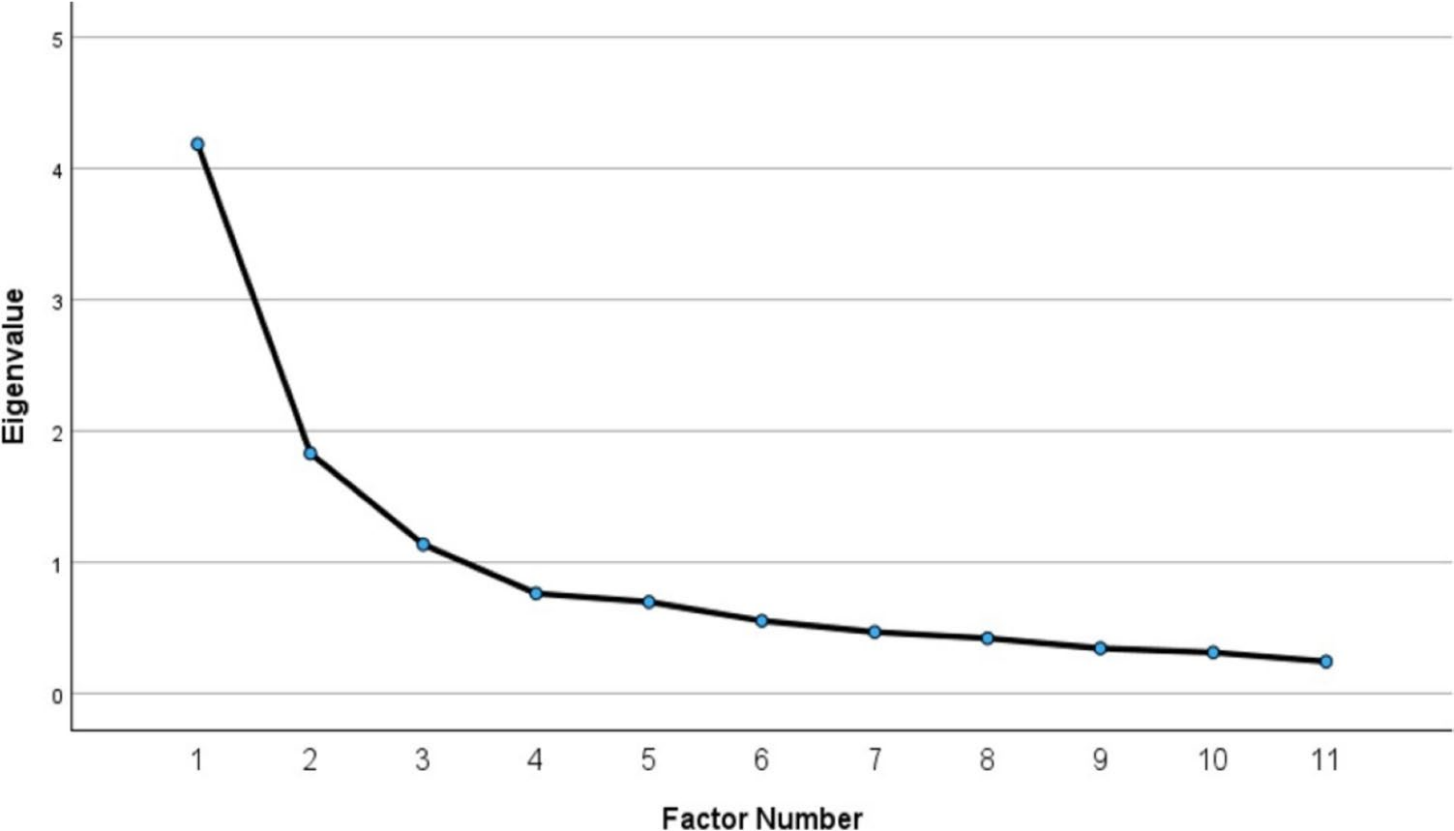
Scree plot of eigenvalues visually suggesting the number of factors to retain

Examination of the scree plot (Fig. 2), parallel analysis (Supplementary Material 4), and eigenvalues (Supplementary Material 5) suggested extracting two to three factors. Iterative exploratory factor analysis (EFA), conducted nine times with rotation to refine the model, also supported a 3-factor solution. On the basis of these four investigative approaches, a 3-factor model was deemed appropriate. Items with factor loadings of > 0.30 were retained, consistent with standard practice for EFA and considered theoretically appropriate for this dataset [18, 24]. The 3-factor model solution provided the most stable factor structure with minimal cross-loadings between items, aligned with existing theory and research, had eigenvalues > 1 for all factors, and all items retained had factor loadings > 0.30. The high loadings indicated the items were strongly related to the factor. EFA results are presented in Table 2.

**Table 2:** EFA 3-factor solution of the final PROM items using Promax oblique rotation

Items	Factor 1	Factor 2	Factor 3	Communalities
Q1. In which area(s) of your body do you experience pain?		0.736		0.583
Q2. Please select items that trigger/worsen your pain		0.673		0.521
Q3. How often do you try to avoid the triggers you indicated in question 2?			0.802	0.705
Q4. What type of sensation(s) best describes your pain?		0.863		0.672
Q6. In the past 4 weeks, how intense was the worst of your pain?	0.659			0.557
Q8. Has your pain interfered with any of the following tasks/activities? ^	0.518	0.469		0.642
Q9. In the past 4 weeks, how often has your mood been affected by pain?	0.631			0.391
Q10. In the past 4 weeks, how often have you had trouble sleeping/had poor sleep because of pain?	0.795			0.582
Q11. How does the pain impact your ability to work (paid/unpaid)?	0.621			0.364
Q12. How much has the pain affected your relationships/social life?	0.647			0.521
Q15. What are some of the ways you relieve and manage the pain?		0.534		0.306
Rotated Sums of Squared Loading^a^	3.183	2.862	1.376	
% Variance Explained*	33.992	12.464	6.664	
% Cumulative Variance^#^	33.992	46.456	53.120	

^ Item cross-loaded onto more than one factor

^a^ Eigenvalues following factor rotation. Rotation Sums of Squared Loadings are reported instead of initial eigenvalues, as they represent the variance explained by each factor in the final rotated solution

^*^ Sub-total percentage of variance explained

^#^ Total percentage of the factors explained

Of the five items removed from the draft PROM, one was automatically excluded by SPSS before the analysis due to SPSS due to missing responses that could not be reliably imputed. The remaining four items were systematically removed because of poor factor loading/cross-loading, impact on model stability (the model became unstable because of the items), and lack of theoretical relevance. The final model consisted of 11 items. The three factors in the model included: HRQoL disruptions from post-pelvic floor procedure pain (factor 1), characteristics and management of post-pelvic floor procedure pain (factor 2), and pain avoidance behaviours (factor 3) (Table 2).

The first factor included six items related to disruptions in HRQoL due to pelvic floor procedure-related pain. The second factor encompassed five items describing the characteristics and management of post-procedural pain, with and without mesh complications. Lastly, the third factor consisted of one item capturing pain avoidance behaviours. Only one item cross-loaded (overlapped) onto more than one factor, suggesting a good discriminant structure among most items (Table 2).

### Structural Validity

Correlation analysis among the factors indicated a moderate positive relationship between factor 1 and factor 2 (*r* = 0.436) (Table 3). Factors 1 and 3 demonstrated a weak positive correlation (*r* = 0.305), whilst factors 2 and 3 had the weakest correlation (*r* = 0.254), suggesting partial independence of pain avoidance behaviours from HRQoL disruption and pain characteristics (Table 3).

**Table 3:** Factor correlation matrix

Factor	1	2	3
1	1.000	0.436	0.305
2	0.436	1.000	0.254
3	0.305	0.254	1.000

The 3-factor EFA model supports structural validity for most items as the items have sufficiently high communality values of > 0.60, meaning they align well with the measured construct (Table 2). In terms of the total variance explained, the 3-factor model accounted for approximately 53% of the differences in responses to items (Table 2). This level of explained variance is considered acceptable and is a strong result, indicative of a good fit given the complexity of pain experiences.

### Internal Consistency

The overall Cronbach’s *α* of the PROM was 0.766, indicating an ‘acceptable’ level of internal consistency [23]. Item-level analysis showed alpha values ranging from 0.711 to 0.767, suggesting that each item contributed meaningfully to the overall reliability of the instrument (Supplementary Material 6).

### Final Set of Items in a Revised PROM

Of the original eight domains, the sixth domain of ‘comorbidities and complications’ was removed during analysis. This was because the first item in the domain was excluded by SPSS due to missing values, and the second item, a follow-up question, was deemed irrelevant and subsequently removed. Following EFA, a final set of 11 items was selected for the new pain-specific PROM, resulting in a short-form version (see Supplementary Material 7). In the final set of items, question one corresponded to domain one (*region of pain*); questions two and three to domain two (*pain triggers*); question four to domain three (*sensation of pain*); question five matched to domain four (*intensity and continuity of pain*); questions six to ten corresponded to the fifth domain of *pain interference*; and question 11 to domain seven of (*pain relief and management*).

## Discussion

This study evaluated the psychometric properties of a new pain-specific PROM through field testing with women from international pelvic mesh support groups. Our factor analysis identified a stable 3-factor structure with strong loadings, leading to the refinement of the preliminary version of the PPPQ—a now shortened, final version comprising 11 items. The analysis revealed three distinct constructs captured by the PPPQ: (1) HRQoL disruptions from post-pelvic floor procedure pain; (2) characteristics and management of post-pelvic floor procedure pain; and (3) pain avoidance behaviours. These factors reflect the complex and multidimensional nature of postoperative pain, consistent with existing literature that conceptualises pelvic floor procedural pain as both a physical and psychosocial experience [25].

Importantly, the 3-factor model demonstrated good structural integrity, with minimal cross-loading and acceptable communalities for most items. While three items (Q9, Q11, Q15) exhibited lower communalities, their values remained within acceptable thresholds (0.30–0.40), indicating potential for further refinement rather than exclusion. The moderate correlation between HRQoL disruption and pain characteristics aligns with existing literature linking pain intensity and persistence with quality of life impairments [26]. In contrast, the weaker associations with avoidance behaviours may suggest that these coping strategies are more individualised and context-dependent, shaped by aspects like psychological makeup, cultural background, or previous medical experiences [27].

It was difficult to draw direct comparisons between the PPPQ and existing pain-related instruments, as no other PROM has been specifically developed to assess pain post-pelvic floor surgery [6]. While several instruments capture aspects of pelvic pain or general pain experiences, they do not fully reflect the unique features of post-pelvic floor procedure pain, unlike the PPPQ [6]. For example, the McGill Pain Questionnaire (MPQ) assesses the multidimensional nature of pain, including sensation, intensity and frequency, but it lacks relevance to pelvic floor surgery as it does not capture visceral (deep somatic) linked to pelvic floor pain or pelvic-specific descriptors, and fails to address location and activity triggers specific to PFDs [6]. The Pelvic Floor Distress Inventory (PFDI-20), widely used in assessing the impact of PFD on quality of life, focuses on symptoms related to prolapse, bladder and bowel function, with limited focus on pain. It does not include questions specific to pelvic floor procedures, but it is also known to be used pre- and postoperatively to evaluate symptom change [6].

Other instruments, such as the Pelvic Pain Impact Questionnaire (PPIQ), examine the impact of pelvic pain; however, they do not capture pelvic floor procedural pain [6]. Generic quality of life measures such as the EQ-5D assess the broad impacts on function and wellbeing, like constructs measured in the PPPQ. Although recent evidence has supported the construct validity of the EQ-5D in patients with chronic pelvic pain, it remains a generic PROM that does not capture pain-specific domains or pelvic floor surgical experiences [6].

In addition to addressing this important content gap, the PPPQ demonstrated good internal consistency, and the item-level analysis supported the contribution of the individual items to the overall construct—pelvic floor procedural pain. Similarly, the structural validity findings of the PROM support the conclusion that the PROM measures what it is intended to measure. The item groups align with the expected constructs, reflecting how pain affects quality of life, the nature of the pain, and an individual’s attempts to manage or avoid it. This suggests that the PROM accurately captures the distinct yet interconnected aspects of pain experienced following a pelvic floor procedure. Compared to existing instruments like the PFDI-20 and others, including the International Consultation on Incontinence Questionnaire Vaginal Symptoms (ICIQ-VS), which reported Cronbach’s alpha values between 0.70 and 0.85, the PPPQ performed within a similar range [28]. Such reliability findings are encouraging given how difficult it is to accurately measure pelvic floor surgery pain [6].

A key strength of this study was the inclusion of an international sample of participants, which enhanced the diversity and real-world relevance of the PROM testing [15]. Additionally, promoting the study on social media platforms enabled participation from women outside the identified support groups, broadening the sample further. However, recruiting primarily through pelvic mesh support groups introduced potential sampling bias and the risk of excluding certain surgical subgroups. Specifically, the sample may not fully represent the broader population of women undergoing pelvic floor procedures (i.e. those receiving native tissue repair). Additional limitations included the inability to conduct a full psychometric assessment, specifically CFA, recommended by COSMIN guidelines following EFA. Furthermore, the present study focused on the analysis of the reduced item set, as factor analysis of the full item pool of 114 items would have required a substantially larger sample. These limitations were beyond the scope of this study [16]. Additionally, no gold-standard validated measure was available for comparison with the new instrument. Another limitation was the use of mean imputation to address missing data, even though the overall proportion of missing responses was small and appeared random. While this approach preserved the full sample for analysis, it may have slightly reduced variability and weakened inter-item correlations, potentially exerting a minor influence on the factor structure and reliability estimates [29].

## Conclusion

This study aimed to undertake a psychometric evaluation of the new pain-specific PROM through field testing. The factor analysis revealed a stable 3-factor structure with strong loadings and led to a refined set of items for the revised pain-specific PROM. Psychometric assessment of the PROM demonstrated good reliability (internal consistency) and validity (structural validity), suggesting that the measure would be suitable for assessing pain in women post-pelvic floor procedure. To further enhance the reliability and validity of the PPPQ, additional psychometric evaluation with a large sample of women is planned. Future steps include undertaking theory-driven, domain-specific CFA with additional model fit indices to examine sensory and affective pain constructs as distinct dimensions, examining test–retest reliability, evaluating responsiveness and sensitivity to change, finalising the instrument, and seeking to patent the PROM for use in clinical care settings (i.e. specialist clinics, hospitals) locally and internationally.

## Supplementary Information

Below is the link to the electronic supplementary material.

Supplementary file1 (DOCX 15 KB)

Supplementary file2 (DOCX 106 KB)

Supplementary file3 (DOCX 19 KB)

Supplementary file4 (DOCX 15 KB)

Supplementary file5 (DOCX 17 KB)

Supplementary file6 (DOCX 17 KB)

Supplementary file7 (DOCX 37 KB)

## Data Availability

Data sharing does not apply to this article as no datasets were generated and/or analysed during the current study.
