# Supplementary material for: Psychometric Assessment of a New Pain-Specific Patient-Reported Outcome Measure for Pelvic Floor Surgery Using Exploratory Factor Analysis
Source: Int Urogynecol J. 2026 Apr 16;37(6):1809–17. doi: 10.1007/s00192-026-06620-9 (PMC13309405; doi:10.1007/s00192-026-06620-9)
Supplement: Supplementary file 1 — Supplementary file1 (DOCX 15 KB) [file 192_2026_6620_MOESM1_ESM.docx]

**Supplementary Material 1: Demographic/Screening Questions**

1. What is your age (years)? ________
2. Please provide the date of your recent pelvic floor procedure (dd/mm/yyyy): __/__ /__
3. Did you develop pain following your pelvic floor procedure?

- YES –

a) How soon after your procedure did you develop pain? Please tick one option.

- - Within 1 week
  - Within 1 month
  - Within 6 months
  - Within 1 year
  - After more than 1 year
- NO
