# Supplementary figures and images for: Psychometric Assessment of a New Pain-Specific Patient-Reported Outcome Measure for Pelvic Floor Surgery Using Exploratory Factor Analysis
Source: Int Urogynecol J. 2026 Apr 16;37(6):1809–17. doi: 10.1007/s00192-026-06620-9 (PMC13309405; doi:10.1007/s00192-026-06620-9)

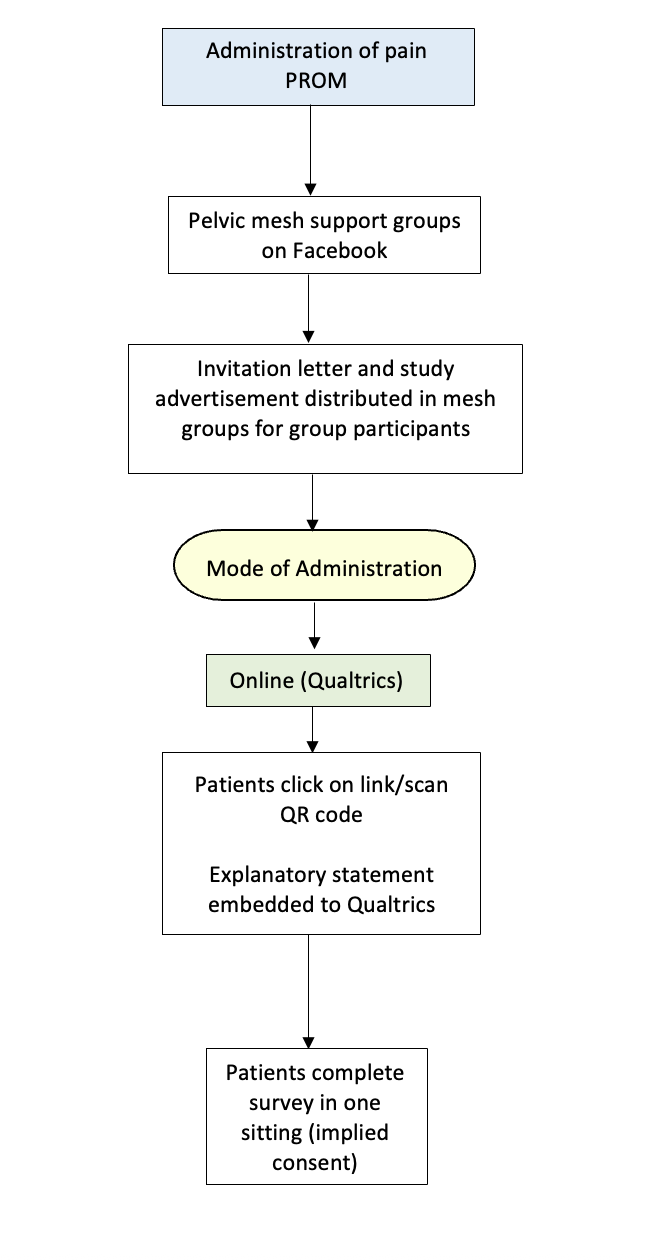
**Supplementary Material 2: PROM Administration Flow Diagram**

Supplement: Supplementary file 2 — Supplementary file2 (DOCX 106 KB) [file 192_2026_6620_MOESM2_ESM.docx]
