# Supplementary material for: Psychometric Assessment of a New Pain-Specific Patient-Reported Outcome Measure for Pelvic Floor Surgery Using Exploratory Factor Analysis
Source: Int Urogynecol J. 2026 Apr 16;37(6):1809–17. doi: 10.1007/s00192-026-06620-9 (PMC13309405; doi:10.1007/s00192-026-06620-9)
Supplement: Supplementary file 3 — Supplementary file3 (DOCX 19 KB) [file 192_2026_6620_MOESM3_ESM.docx]

**Supplementary Material 3: SPSS Output for KMO and Bartlett’s Test, and Correlation Matrix**

| **KMO and Bartlett's Test** | | |
| --- | --- | --- |
| Kaiser-Meyer-Olkin Measure of Sampling Adequacy. | | .796 |
| Bartlett's Test of Sphericity | Approx. Chi-Square | 320.288 |
|  | df | 55 |
|  | Sig. | <.001 |

*KMO index* is an indication of whether makes sense to conduct EFA on the set of items (Williams et al, *Australasian Journal of Paramedicine*, 2010)

*Bartlett’s Test of Sphericity* determines if the correlation matrix is significantly different from an identify matrix (matrix where variables are uncorrelated). For factor analysis to be suitable, the test should be significant (p <0.05) (Kaiser, *Psychometrika*, 1974)

| **Correlation Matrix^a^** | | | | | | | | | | | | |
| --- | --- | --- | --- | --- | --- | --- | --- | --- | --- | --- | --- | --- |
|  | | Q1Dom1 | Q2Dom2 | Q3Dom2 | Q4Dom3 | Q6Dom4 | Q8Dom5 | Q9Dom5 | Q10Dom5 | Q11Dom5 | Q12Dom5 | Q15Dom7 |
| Correlation | Q1Dom1 | 1.000 | .577 | .333 | .614 | .249 | .403 | .047 | .217 | .192 | .236 | .308 |
|  | Q2Dom2 | .577 | 1.000 | .235 | .575 | .252 | .442 | .199 | .244 | .255 | .321 | .339 |
|  | Q3Dom2 | .333 | .235 | 1.000 | .224 | .381 | .207 | .266 | .177 | .049 | .419 | -.005 |
|  | Q4Dom3 | .614 | .575 | .224 | 1.000 | .200 | .485 | .124 | .131 | .053 | .126 | .379 |
|  | Q6Dom4 | .249 | .252 | .381 | .200 | 1.000 | .522 | .504 | .547 | .304 | .510 | .074 |
|  | Q8Dom5 | .403 | .442 | .207 | .485 | .522 | 1.000 | .336 | .482 | .426 | .434 | .447 |
|  | Q9Dom5 | .047 | .199 | .266 | .124 | .504 | .336 | 1.000 | .499 | .317 | .383 | .045 |
|  | Q10Dom5 | .217 | .244 | .177 | .131 | .547 | .482 | .499 | 1.000 | .437 | .510 | .163 |
|  | Q11Dom5 | .192 | .255 | .049 | .053 | .304 | .426 | .317 | .437 | 1.000 | .469 | .135 |
|  | Q12Dom5 | .236 | .321 | .419 | .126 | .510 | .434 | .383 | .510 | .469 | 1.000 | .199 |
|  | Q15Dom7 | .308 | .339 | -.005 | .379 | .074 | .447 | .045 | .163 | .135 | .199 | 1.000 |
| a. Determinant = .014 | | | | | | | | | | | | |
