# Supplementary material for: Psychometric Assessment of a New Pain-Specific Patient-Reported Outcome Measure for Pelvic Floor Surgery Using Exploratory Factor Analysis
Source: Int Urogynecol J. 2026 Apr 16;37(6):1809–17. doi: 10.1007/s00192-026-06620-9 (PMC13309405; doi:10.1007/s00192-026-06620-9)
Supplement: Supplementary file 4 — Supplementary file4 (DOCX 15 KB) [file 192_2026_6620_MOESM4_ESM.docx]

**Supplementary Material 4: SPSS Output for Parallel Analysis**

Run MATRIX procedure:

PARALLEL ANALYSIS:

PAF/Common Factor Analysis & Random Normal Data Generation

Specifications for this Run:

Ncases 74

Nvars 15

Ndatsets 1000

Percent 95

Raw Data Eigenvalues, & Mean & Percentile Random Data Eigenvalues

Root Raw Data Means Prcntyle

1.000000 3.631154 1.083930 1.318853

2.000000 1.583565 .854370 1.034041

3.000000 .804484 .691148 .834415

4.000000 .761911 .549627 .668375

5.000000 .370507 .424544 .529086

6.000000 .271627 .313642 .405071

7.000000 .148904 .209423 .299487

8.000000 .063505 .114517 .196288

9.000000 -.048411 .027659 .102113

10.000000 -.079195 -.054849 .010162

11.000000 -.123758 -.129694 -.072233

12.000000 -.144482 -.202160 -.153084

13.000000 -.184844 -.268755 -.224823

14.000000 -.279599 -.333912 -.291785

15.000000 -.319430 -.403401 -.360020

------ END MATRIX -----

***Parallel analysis output interpretation:*** Eigenvalues for the first two factors exceed 95th percentiles. Analysis suggested the extraction of two factors.
