# Supplementary material for: Psychometric Assessment of a New Pain-Specific Patient-Reported Outcome Measure for Pelvic Floor Surgery Using Exploratory Factor Analysis
Source: Int Urogynecol J. 2026 Apr 16;37(6):1809–17. doi: 10.1007/s00192-026-06620-9 (PMC13309405; doi:10.1007/s00192-026-06620-9)
Supplement: Supplementary file 5 — Supplementary file5 (DOCX 17 KB) [file 192_2026_6620_MOESM5_ESM.docx]

**Supplementary Material 5: SPSS Output for Eigenvalues**

| **Total Variance Explained** | | | | | | | |
| --- | --- | --- | --- | --- | --- | --- | --- |
| Factor | Initial Eigenvalues | | | Extraction Sums of Squared Loadings | | | Rotation Sums of Squared Loadings^a^ |
|  | Total | % of Variance | Cumulative % | Total | % of Variance | Cumulative % | Total |
| 1 | 4.190 | 38.088 | 38.088 | 3.739 | 33.992 | 33.992 | 3.183 |
| 2 | 1.835 | 16.681 | 54.770 | 1.371 | 12.464 | 46.456 | 2.862 |
| 3 | 1.140 | 10.366 | 65.136 | .733 | 6.664 | 53.120 | 1.376 |
| 4 | .767 | 6.969 | 72.105 |  |  |  |  |
| 5 | .702 | 6.379 | 78.484 |  |  |  |  |
| 6 | .558 | 5.072 | 83.555 |  |  |  |  |
| 7 | .472 | 4.294 | 87.849 |  |  |  |  |
| 8 | .425 | 3.860 | 91.708 |  |  |  |  |
| 9 | .348 | 3.166 | 94.875 |  |  |  |  |
| 10 | .317 | 2.879 | 97.754 |  |  |  |  |
| 11 | .247 | 2.246 | 100.000 |  |  |  |  |
| Extraction Method: Principal Axis Factoring. | | | | | | | |
| a. When factors are correlated, sums of squared loadings cannot be added to obtain a total variance. | | | | | | | |
