# Supplementary material for: Psychometric Assessment of a New Pain-Specific Patient-Reported Outcome Measure for Pelvic Floor Surgery Using Exploratory Factor Analysis
Source: Int Urogynecol J. 2026 Apr 16;37(6):1809–17. doi: 10.1007/s00192-026-06620-9 (PMC13309405; doi:10.1007/s00192-026-06620-9)
Supplement: Supplementary file 6 — Supplementary file6 (DOCX 17 KB) [file 192_2026_6620_MOESM6_ESM.docx]

**Supplementary Material 6: SPSS Output for Internal Consistency**

| **Reliability Statistics** | | |
| --- | --- | --- |
| Cronbach's Alpha | Cronbach's Alpha Based on Standardized Items | N of Items |
| .766 | .801 | 11 |

| **Item-Total Statistics** | | | | | |
| --- | --- | --- | --- | --- | --- |
|  | Scale Mean if Item Deleted | Scale Variance if Item Deleted | Corrected Item-Total Correlation | Squared Multiple Correlation | Cronbach's Alpha if Item Deleted |
| Q1Dom1 | 50.36 | 159.869 | .614 | .542 | .718 |
| Q2Dom2 | 51.53 | 163.343 | .657 | .480 | .711 |
| Q3Dom2 | 55.62 | 216.603 | .239 | .327 | .766 |
| Q4Dom3 | 49.63 | 145.042 | .598 | .560 | .727 |
| Q6Dom4 | 55.56 | 214.639 | .383 | .442 | .762 |
| Q8Dom5 | 46.18 | 137.967 | .633 | .546 | .723 |
| Q9Dom5 | 55.94 | 217.204 | .223 | .286 | .767 |
| Q10Dom5 | 55.71 | 211.899 | .391 | .383 | .759 |
| Q11Dom5 | 55.73 | 211.160 | .301 | .313 | .761 |
| Q12Dom5 | 55.19 | 213.092 | .406 | .448 | .760 |
| Q15Dom7 | 56.00 | 195.792 | .497 | .348 | .743 |
