# Supplementary material for: Psychometric Assessment of a New Pain-Specific Patient-Reported Outcome Measure for Pelvic Floor Surgery Using Exploratory Factor Analysis
Source: Int Urogynecol J. 2026 Apr 16;37(6):1809–17. doi: 10.1007/s00192-026-06620-9 (PMC13309405; doi:10.1007/s00192-026-06620-9)
Supplement: Supplementary file 7 — Supplementary file7 (DOCX 37 KB) [file 192_2026_6620_MOESM7_ESM.docx]

**Supplementary Material 7: Pain-Specific PROM following psychometric evaluation (n=11 items)**

**POST-PELVIC FLOOR PROCEDURE PAIN QUESTIONNAIRE (PPPQ)**

Today’s date (dd/mm/yyyy):___/___/_____ Age (years):______

| **DOMAIN 1: REGION OF PAIN** |
| --- |
| **In which area(s) of your body do you experience pain?** *Tick all that apply.*   - 1 = Upper leg (e.g. thigh) - 1 = Buttock - 1 = Genitals (vulva, vagina) - 1 = Pelvis - 1 = Pubic bone - 1 = Hip - 1 = Bladder - 1 = Bowel - 1 = Skin/tissue - 1 = Lower leg (e.g. calf) - 1 = Foot - 1 = Groin - 1 = Upper torso - 1 = Upper arm and shoulder - 1 = Forearm - 1 = Lower abdomen - 1 = Back and spine |
| **DOMAIN 2: PAIN TRIGGERS** |
| **Please select items that trigger/worsen your pain.** *Tick all that apply.*   - 1 = Stress/Anxiety - 1 = Full bowel - 1 = Urinating - 1 = Having a bowel movement - 1 = Temperature - 1 = Intercourse - 1 = Orgasm - 1 = Menstrual cramps - 1 = Other not listed here - 1 = Heightened emotions - 1 = Trauma (physical, psychological) - 1 = Activity/Exercise (i.e. movement, physical activity) - 1 = Cessation of clinical exercise/therapy - 1 = Posture (lying down, sitting, standing) - 1 = Food and beverages - 1 = Full bladder |
| **How often do you try to avoid the triggers you indicated in question 2?** *Tick* ***one*** *box only.*   - 0 = Never - 1 = Rarely - 2 = Sometimes - 3 = Often - 4 = Always |
| **DOMAIN 3: SENSATION OF PAIN** |
| **What type of sensation(s) best describes your pain?** *Tick all that apply.*   - 1 = Sharp - 1 = Dragging - 1 = Pulling - 1 = Ripping - 1 = Cutting - 1 = Pressure - 1 = Burning/stinging - 1 = Numbness - 1 = Tingling - 1 = Electric shock - 1 = Stabbing - 1 = Poking - 1 = Aching - 1 = Cramping - 1 = Spasm - 1 = Throbbing - 1 = Pulsating - 1 = Dull |
| **DOMAIN 4: INTENSITY OF PAIN** |
| **In the past 4 weeks, how intense was the worst of your pain?** *Tick* ***one*** *box only.*   - 0 = Not intense (did not have pain/sensations) - 1 = Mild - 2 = Moderate - 3 = Severe - 4 = Very severe |
| **DOMAIN 5: PAIN INTERFERENCE** |
| **Has your pain interfered with any of the following tasks/activities?** *Tick all that apply.*   - 0 = None - 1 = Driving - 1 = Work - 1 = Studying - 1 = Volunteering - 1 = Sleeping - 1 = Being intimate - 1 = Urinating - 1 = Having a bowel movement - 1 = Bathing/showering - 1 = Hygiene/self-care - 1 = Daily chores - 1 = Looking after family - 1 = Walking - 1 = Posture (sitting, standing) - 1 = Physical activity, sport, exercise - 1 = Hobbies and leisure - 1 = Socialising - 1 = Attending medical appointments - 1 = Travel |
| **In the past 4 weeks, how often has your mood been affected by pain?** *Tick* ***one*** *box only.*   - 0 = None of the time - 1 = A little of the time - 2 = Some of the time - 3 = Most of the time - 4 = All of the time |
| **In the past 4 weeks, how often have you had trouble sleeping/had poor sleep because of pain?** *Tick* ***one*** *box only.*   - 0 = None of the time - 1 = A little of the time - 2 = Some of the time - 3 = Most of the time - 4 = All of the time |
| **How does the pain impact your ability to work (paid/unpaid)?** *Tick* ***one*** *box only.*   - 0 = None – able to work at full capacity - 1 = Had to reduce work hours - 2 = Had to reduce the number of days working (move to casual/part-time) - 3 = Had to change career - 4 = Had to stop working completely |
| **How much has the pain affected your relationships/social life?** *Tick* ***one*** *box only.*   - 0 = Not at all - 1 = A little - 2 = Somewhat - 3 = Moderately - 4 = A great deal |
| **DOMAIN 6: PAIN RELIEF AND MANAGEMENT** |
| **What are some of the ways you relieve and manage the pain?** *Tick all that apply.*   - 1 = Medications/creams/ointments - 1 = Natural remedies - 1 = Massage - 1 = Meditation - 1 = Physiotherapy - 1 = Psychological therapy - 1 = Acupuncture |
